# Molecular characterization of lumpy skin disease virus (LSDV) emerged in Bangladesh reveals unique genetic features compared to contemporary field strains

**DOI:** 10.1186/s12917-021-02751-x

**Published:** 2021-01-29

**Authors:** Shukes Chandra Badhy, Mohammad Golam Azam Chowdhury, Tirumala Bharani Kumar Settypalli, Giovanni Cattoli, Charles Euloge Lamien, Mohammad Aflak Uddin Fakir, Shamima Akter, Mozaffar Goni Osmani, Faisol Talukdar, Noorjahan Begum, Izhar Ahmed Khan, Md Bazlur Rashid, Mohammad Sadekuzzaman

**Affiliations:** 1 Central Disease Investigation Laboratory (CDIL), 48, KaziAlauddin Road, Dhaka, People’s Republic of Bangladesh; 2 Department of Livestock Services, Dhaka, People’s Republic of Bangladesh; 3 Animal Production and Health Laboratory, Joint FAO/IAEA Division of Nuclear Techniques in Food and Agriculture, Department of Nuclear Sciences and Applications, International Atomic Energy Agency, Wagramer Strasse 5, P.O. Box 100, A-1400 Vienna, Austria

**Keywords:** Lumpy skin disease virus;Capripoxvirus;RPO30, GPCR, EEV glycoprotein, Bangladesh

## Abstract

**Background:**

Lumpy skin disease (LSD) is a contagious viral disease of cattle caused by lumpy skin disease virus (LSDV). LSD has recently spread in Asia following outbreaks in the Middle East and Europe. The disease emerged in Bangladesh in July 2019 in the Chattogram district, then rapidly spread throughout the entire country. We investigated six LSD outbreaks in Bangladesh to record the clinical signs and collect samples for diagnostic confirmation. Furthermore, we performed the molecular characterization of Bangladesh isolates, analyzing the full RPO30 and GPCR genes and the partial EEV glycoprotein gene.

**Results:**

Clinical observations revealed common LSD clinical signs in the affected cattle. PCR and real-time PCR, showed the presence of the LSDV genome in samples from all six districts. Phylogenetic analysis and detailed inspection of multiple sequence alignments revealed that Bangladesh isolates differ from common LSDV field isolates encountered in Africa, the Middle East, and Europe, as well as newly emerged LSDV variants in Russia and China. Instead, they were closely related to LSDV KSGP-0240, LSDV NI2490, and LSDV Kenya.

**Conclusions:**

These results show the importance of continuous monitoring and characterization of circulating strains and the need to continually refine the strategies for differentiating vaccine strains from field viruses.

**Supplementary Information:**

The online version contains supplementary material available at 10.1186/s12917-021-02751-x.

## Background

Lumpy skin disease (LSD) is a viral disease of cattle, caused by lumpy skin disease virus (LSDV) within the genus *Capripoxvirus*, family *Poxviridae*. The genus *Capripoxvirus* also comprises goatpox virus (GTPV) and sheeppox virus (SPPV). LSD is a notifiable disease by the World Organization for Animal Health (OIE) because of its potential rapid spread and substantial economic importance.

LSDV has a limited host range and does not infect non-ruminant hosts [1]. Both sexes and all ages of cattle breeds are susceptible to LSDV. However, younger animals may be more susceptible to the severe form of the disease [2]. Even in close contact with infected cattle, sheep and goats never developed LSD [3], with one noted exception, LSDVKSGP-0240 also known as LSDV KS1.

There is a significant variation of clinical signs with LSDV infections ranging from subclinical infection to death [4]. The main clinical signs include fever, the appearance of nodules in the skin, lesions in the mouth, pharynx, enlarged superficial lymph nodes, edema in the skin, and sometimes death [3–5]. There is an initial incubation period of 6 to 9 days during acute cases followed by a fever that may exceed 41 °C [6].

LSD is one of the most economically significant viral diseases of cattle because of the loss of production, permanent damage of hides, infertility, and death. Although the mortality rate is usually less than 10%, the disease morbidity rate can be as high as 100% [7].

For many years, the LSDV genome appeared to be stable. Indeed, following its first description in Zambia in 1929 [8], LSDV field isolates recovered for decades in Africa showed only minor genomic differences [9–12]. As the disease further spread into the Middle East from 2012 [13] and Europe in 2015 [7], the recovered LSDV field isolates showed little variability to contemporary African LSDV field isolates [14–16]. This genetic stability was exploited for the differentiation of LSDV live attenuated vaccines from contemporary field isolates [16–21].

However, this dynamic has shifted following the discovery of field LSDVs in Russia in 2017 and 2019 showing vaccine-like profiles [22–25]. Some of these LSDV variants, presented a 12-nucleotide insertion in the GPCR gene, like vaccine strains, and others presented a27-nucleotide deletion, similar to the LSDV Neethling vaccine strain, in the ORF LSDV 126. The authors attributed these variants’ emergence to recombination events between the Neethling vaccine strain and field isolates [23]. This has prompted researchers [26] to question the relevance of current strategies to differentiate LSDV vaccine strains from viral field strains. Similarly, the LSDV strains involved in the outbreaks in China present the GPCR profile of LSDV vaccines with the 27-nucleotide insertion in their EEV glycoprotein gene. Interestingly, Kononova et al. [27], also showed in vitro and in vivo that the recombinant LSDVs could induce more severe disease than the typical field isolates.

The increased variability of LSDV in recent years makes it crucial to adapt the molecular DIVA strategies based on the knowledge of the circulating strain of LSDV. This requires the constant monitoring and characterization of LSDV field isolates.

Several PCR, real-time PCR, and HRM based methods are available for the detection of the LSDV genome, [12, 14, 28–36] and molecular epidemiological studies of LSDV rely on analyzing various genomic regions, such as the GPCR, the RPO30, the P32, and the EEV glycoprotein genes [11, 12, 17–19, 37].

On September 15, 2019, Bangladesh notified to OIE the first outbreak of LSD in the country. The disease started in July 2019 in the Southeast (Chattogram district) of the country, then progressively spread throughout the country. Because of the wide distribution and large cattle population in Bangladesh, LSD is now one of the most economically important emerging livestock disease in Bangladesh.

This study aimed to investigate and confirm the recent outbreaks and provide LSDV molecular characterization in different regions in Bangladesh.

## Results

### Outbreak investigation

**Fig. 1:**
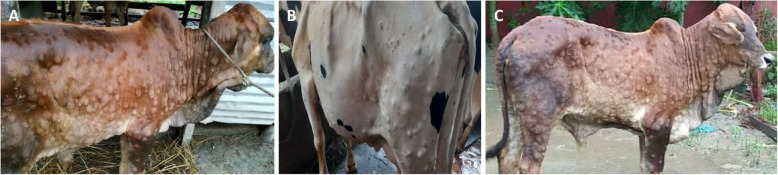
Skin lesions characteristics of lumpy skin disease in 3 animals in Bangladesh. The generalized circumscribed active nodular skin lesions covering the entire body are visible. Source: own

**Fig. 2:**
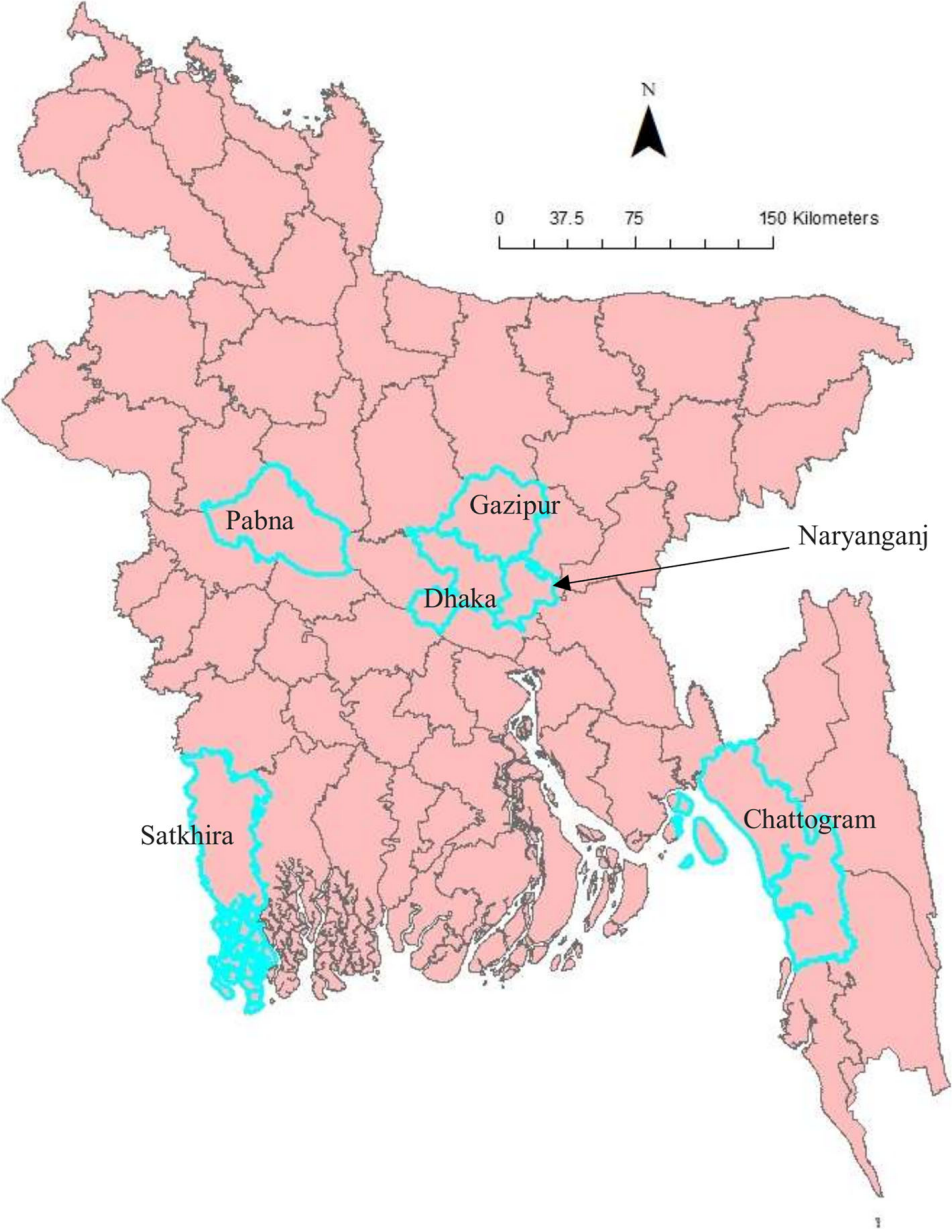
Map of Bangladesh showing the sample collection area. The map is an own creation using Arc GIS software version 13.2

All affected cattle in different districts in Bangladesh (Chottogram, Dhaka, Gazipur, Narayanganj, Pabna, and Satkhira) showed the following common clinical signs: fever (40–41 °C), depression, loss of appetite, nasal and ocular discharges, salivation, circumscribed nodules with different sizes on the skin covering their head, neck, trunk, perineum, udder, and teats. Figure 1 illustrates the skin lesions of affected cattle. In many infected animals, the necrotic nodules were ulcerated and formed deep scabs (sitfast). Moreover, a decrease in body weight and reduced milk production were prominent signs observed in cattle affected by LSD. The total cattle population, reported morbidities and mortalities in the six districts of this study (Fig. 2) are sumarised in Table 1.

**Table 1:** Estimated morbidity and mortality in sample collection area. The total cattle population, number of reported cases and mortality in six districts are shown

Location	Number of cattle	Number of reported LSD cases	Number of reported LSD death cases^a^	Morbidity (%)	Mortality (%)
Chattogram	796,000	185,172	04	23	0.002
Dhaka	226,000	473	01	0.21	0.0004
Naryanganj	79,000	691	0	0.87	0
Gazipur	322,000	4573	01	1.42	0.0003
Satkhira	393,000	242	0	0.06	0
Pabna	759,000	416	0	0.05	0

^a^Source: Epidemiology Unit -Department of Livestock Services, Bangladesh

### Molecular detection of LSDV

**Fig. 3:**
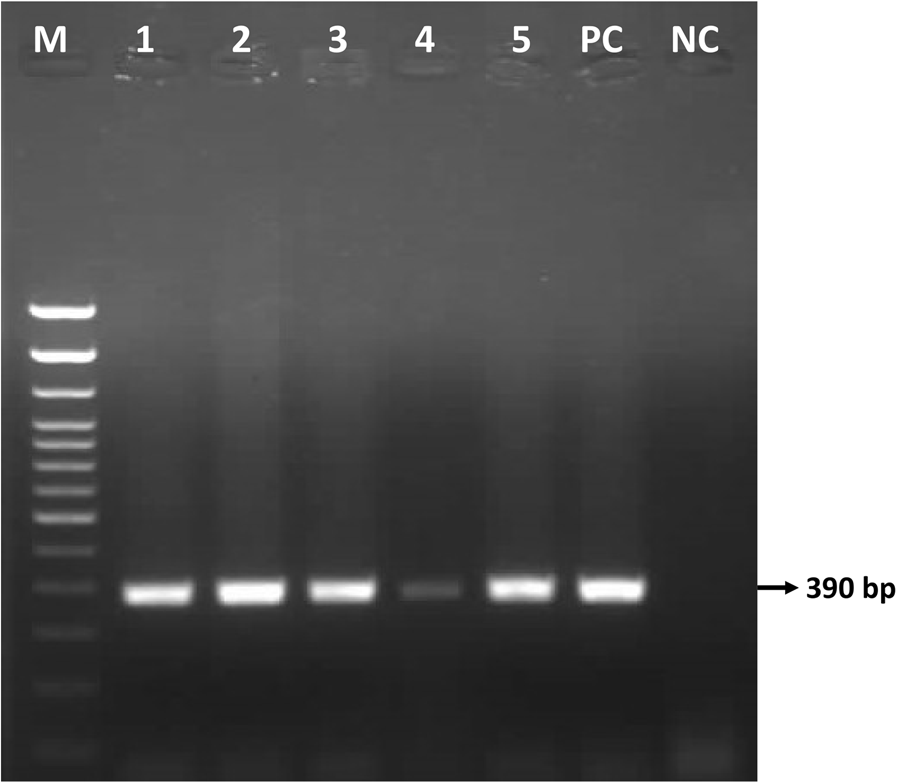
Agarose gel electrophoresis showing the 390 bp amplicon of P32 gene for selected samples of Bangladesh. Lane M: 100 bp DNA ladder, Lane 1–5: LSDV field samples, Lane PC: positive control, lane NC: negative control

Gel electrophoresis of the P32 amplicons showed a 390 bp product in all fifty (50) samples collected in six districts, as illustrated for selected samples in Fig. 3.

The real-time PCR result confirmed capripoxvirus DNA in all samples. Six representative samples, one per district with a Cq value between 19.17 and 25.31, were selected for sequencing (Table 2).

**Table 2:** Samples analyzed for PCR and sequencing. Samples were collected from six locations affected by affected by lumpy skin disease in Bangladesh in 2019

Outbreak date	Location	Number of samples collected	Types of samples	No of PCR positive samples	Number of amplicons sequenced		
					RPO30 gene	GPCR gene	EEV glycoprotein
July, 2019	Chattogram	12	Skin tissue, blood, saliva. Skin scab	12	1	1	1
July, 2019	Dhaka	10	Skin tissue, skin scab	10	1	1	1
August, 2019	Naryanganj	8	Skin tissue, skin scab	8	1	1	1
August, 2019	Gazipur	8	Skin tissue, skin scab	8	1	1	1
September, 2019	Satkhira	5	Skin tissue, skin scab	5	1	0	1
September, 2019	Pabna	7	Skin tissue, skin scab	7	1	1	1

### Amplification and sequencing of the RPO30, GPCR, and EEV glycoprotein genes

We have successfully amplified and sequenced two fragments for the RPO30 gene (554 bp and 520 bp) in 6 samples and three for the GPCR (617 bp, 603 bp, and 684 bp) in 5 samples out of 6. We also amplified and sequenced the partial EEV glycoprotein gene in 6 samples. The complete RPO30 and GPCR genes and the partial EEV glycoprotein gene sequences were submitted to the GenBank database under accession numbers MT448690 to MT448701.

### Phylogenetic analysis

**Fig. 4:**
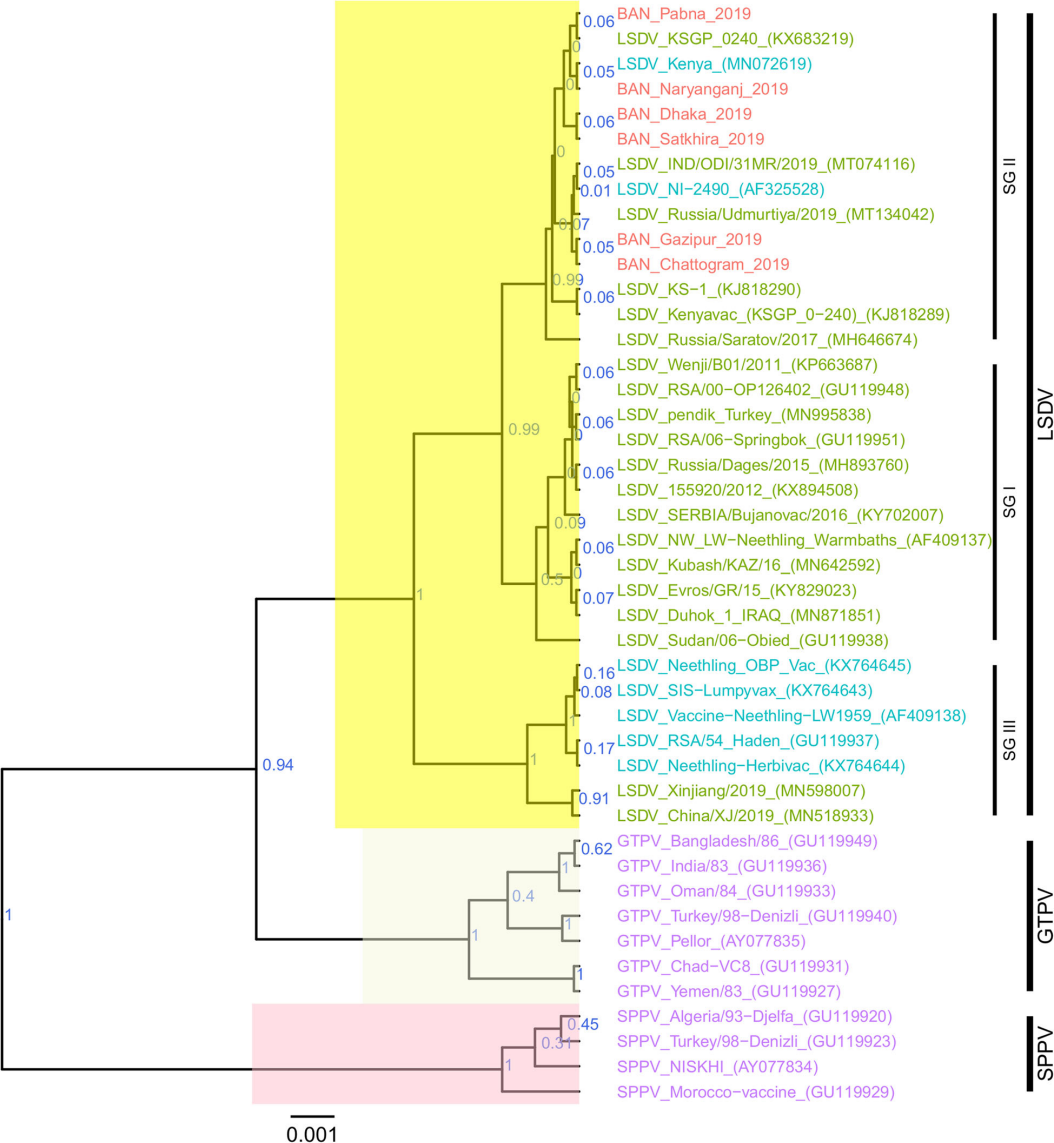
Maximum clade credibility (MCC) tree based on the complete RPO30 complete gene sequences of capripoxviruses. The posterior probabilities are plotted as respective nodes labels. LSDVs from Bangladesh are highlighted in red and reference sequences are represented with their accession numbers

**Fig. 5:**
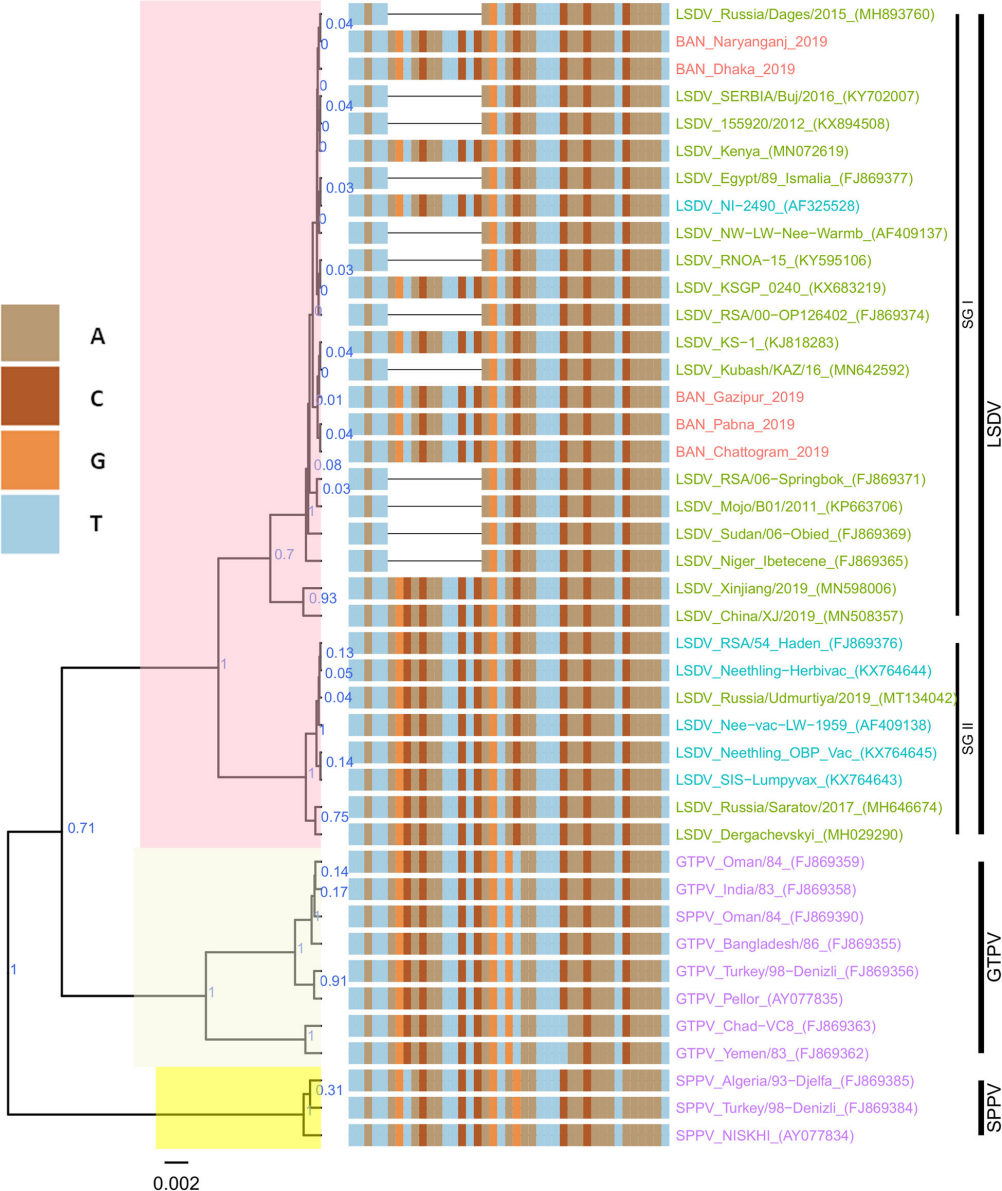
Maximum clade credibility (MCC) tree based on the complete GPCR gene sequences of Capripoxviruses, plotted together with multiple sequence alignment. Only the portion of the alignment between positions 80 and 120 is shown. The posterior probabilities are plotted as respective nodes labels. LSDVs from Bangladesh are highlighted in red and reference sequences are represented with their accession numbers

For each of the targeted genes, the sequences of the Bangladesh LSDVs showed 100% identity among each other. On the phylogenetic trees for both RPO30 (Fig. 4) and GPCR (Fig. 5), all the Bangladesh LSDVs clustered together.

On the RPO30 tree (Fig. 4), Bangladesh isolates clustered within subgroup I, tightly with LSDV KSGP 0240 (KX683219), known as LSDV KS1, LSDV NI-2490 (AF325528), Indian LSDV field isolates, and two recombinant LSDV field isolates from Russia, LSDV Russia/Udmurtiya/2019 (MT134042), and LSDV Russia/Saratov/2017 (MH646674). The commonly circulating field isolates from Africa, the Middle East, and Europe are segregated from the Bangladesh isolates, clustering within subgroup II. A third subgroup contained mainly LSDV Neethling derived vaccine strains, the historical field LSDV RSA/54 Haden, and the LSDV field isolates from China. On the GPCR tree (Fig. 5), there were only two sub-groups. Bangladesh LSDV isolates clustered within subgroup I, together with LSDV SGP_O-240 (KJ818288), LSDV NI-2490 (AF325528), LSDV Kenya (MN072619), common LSDV field isolates from Africa, the Middle East, and Europe, LSDV Xinjiang/2019 (MN598006), and LSDV China/XJ/2019(MN508357) from China. The second subgroup of the GPCR consisted of LSDV Neethling derived vaccines, LSDV RSA/54 Haden (FJ869376), and three recombinant LSDVs from Russia: LSDV Russia/Udmurtiya/2019 (MT134042), LSDV Russia/Saratov/2017 (MH646674) and LSDV Dergachevskyi (MH029290). The multiple sequence alignments of the GPCR gene showed that the Bangladesh LSDV contained the 12-nucleotide insertion (Fig. 5). This 12-nucleotide insertion is also present in the two common LSDV vaccine strains (LSDV KSGP 0240 and LSDV Neethling) and a few historical field isolates (collected before 1960) such as LSDV NI-2490 (AF325528), LSDV Kenya (MN072619), and LSDV RSA/54Haden (GU119937). This insertion is also present in recombinant LSDVs from Russia (LSDV Russia/Udmurtiya/2019, LSDV Russia/Saratov/2017, and LSDV Dergachevskyi) and in recent LSDV isolates from China.

**Fig. 6:**
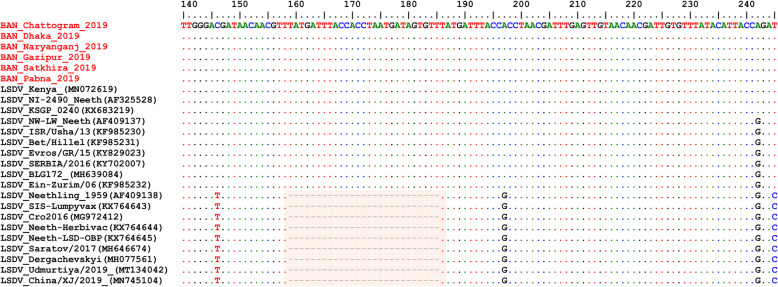
Multiple sequence alignments of the partial nucleotide sequences of the of EEV glycoprotein gene. LSDVs from Bangladesh were aligned with representative LSDVs’ sequences retrieved from GenBank. A unique sequence signature of 27-nucleotide only in LSDV Neethling like viruses is highlighted in the box. Identical nucleotides are indicated with dots

Alignment of the EEV glycoprotein gene sequence showed a 27-nucleotide insertion in all LSDVs from Bangladesh (Fig. 6), which is characteristic of common field isolates and also present in the LSDV KSGP-0240 derived vaccines and historical LSDVs, LSDV NI2490 (1958) and LSDV Kenya (1950), both from Kenya.

Taken together, the analyses of all three targets suggest that the Bangladesh LSDVs were more related to LSDV KSGP-0240, LSDV NI-2490, and LSDV Kenya. They differed from all recent LSDV field isolates, including the LSDVs from China and the recombinant LSDVs described in Russia, and LSDV Neethling vaccine strain.

## Discussion

The diagnosis of LSD was confirmed by real time PCR and the viruses in samples collected from outbreaks between July and September 2019 in Bangladesh were molecularly characterized.

LSD emerged in Bangladesh in July 2019, hitting the district of Chattogram, before quickly spreading to Dhaka, Naryanganj, Gazipur, Satkhira, and Pabna regions, between July and September 2019 [38]. The affected cattle exhibited common clinical manifestations of LSD in cattle, including nasal and ocular discharges and skin lesions [39]. The epidemiology unit data shows a very high incidence of LSD in the Chattogram province compared to other regions in the country. A plausible explanation is that Chattogram, a port city of Bangladesh, is a major coastal city and financial center in southeastern Bangladesh, with more cattle movement due to trade. It was also the first affected province.

The Bangladesh LSDVs presented the 12-nucleotide insertion found in the GPCR gene of LSDV KSGP-0240, LSDV Neethling vaccines, and a few historical LSDVs such as the LSDV NI2490 and LSDV Kenya, LSDVs from China and the recombinant LSDVs described in Russia. The presence of this 12-nucleotide insertion makes them different from commonly circulating field LSDVs encountered in Africa, Europe, and the Middle East [11, 16, 19, 24, 40]. However, a 27-nucleotide insertion in the EEV glycoprotein gene of Bangladesh isolates, and the RPO30 and GPCR gene trees’ analysis differentiated them from LSDV Neethling derived vaccines. This 27-nucleotide insertion in the EEV glycoprotein also makes them different from Chinese LSDV isolates as well as from the recombinant LSDVs described in Russia in recent years.

A close inspection of the sequence alignment of the EEV glycoprotein, the GPCR, and RPO30 genes showed 100% identity to the LSDV KSGP 0240, LSDV NI2490, and LSDV Kenya at the nucleotide level. These features make them unique, as the commonly circulating LSDV isolates have not demonstrated that level of closeness to LSDV KSGP 0240, LSDV NI2490, and LSDV Kenya. It is worth noting that the Bangladesh LSDV RPO30 sequence was 100% identical with the Indian isolate, however, as no GPCR and EEV glycoprotein gene sequences were available from India, it was not possible to extend the comparison.

The existence of vaccine-like field isolates with mixed characteristics between common field viruses and the LSDV Neethling vaccine has been reported in Russia [24]. A more recent report described a field LSDV isolate in Kurgan, Russia, exhibiting similarities to LSDV KSGP 0240 and LSDV NI2490 based on the analysis of GPCR and RPO30 gene fragments [26]. Although the complete RPO30 and GPCR sequences were not available for a full comparison, we noticed a nucleotide difference between the partial RPO30 sequence of the Kurgan isolate and those of this study. Our findings support the circulation of LSDV KS1 or LSDV NI2490-like virus in the field.

How such a virus has emerged suddenly in Bangladesh remains unknown. An extensive characterization of LSDV in neighboring countries could help resolve the emergence of these isolates.

Previous studies showed that the use of the LSDV KSGP 0240 for vaccination could lead to the appearance of generalized lesions [15, 41]. The lesions in cattle in Bangladesh showed pathogenic virus-like lesions, especially the presence of deep “sit fast,” not usually observed with KSGP 0240-induced disease [15]. It is also worth noting that Bangladesh was not vaccinating cattle against LSD before these outbreaks but later started vaccination using a goat poxvirus strain. Therefore, it is unlikely that the administration of a good quality LSDV KSGP 0240 vaccine caused these outbreaks. Furthermore, LSDV KSGP 0240-induced disease manifests only as an adverse reaction in vaccinated animals and shows no signs of animal to animal spread [15, 41].

Historically, viruses resembling LSDV KSGP 0240, the LSDV NI2490 (1958), and LSDV Kenya (1950, but sequenced only recently), caused LSD outbreaks in Kenya [10]; however, these viruses were never detected in subsequent LSDV epidemics in Africa, the Middle East, and Europe. Whether Bangladesh isolates, and presumably those described in Kurgan, Russia, relate to LSDV NI2490 and LSDV Kenya is unclear. Further investigation through full genome sequencing is warranted, as none of the three targeted genes of this study could provide differentiation between LSDV KSGP-0240 and LSDV NI2490.

The reason for the emergence of such LSDV variants remains uncertain. However, recent reports from Russia suggest the possibility of recombination events [24].

## Conclusions

In conclusion, using a multi-targeted approach,we discovered that the viruses causing outbreaks in Bangladesh were different from common contemporary LSDV field isolates circulating worldwide, including the Chinese isolates and the recombinant LSDVs described between 2017 and 2019 in Russia. Full genome analysis will elucidate whether these viruses are LSDV KSGP-0240 or LSDV NI2490/LSDV Kenya. This study highlights the importance of continuous monitoring and characterization of circulating strains and the need to continually refine the strategies for differentiating vaccine strains from field viruses.

## Methods

### Outbreak investigations and sample collection

Fifty (50) biopsies of skin nodules were collected from 6 different districts of Bangladesh (Fig. 2) between July and September 2019. Table 2 shows the location, source, and collection period of LSDV samples. The samples were collected aseptically and transported in a cool box to the Central Disease Investigation Laboratory (CDIL) at Dhaka, Bangladesh. Samples were stored at − 80 °C for further processing.

### Sample preparation and DNA extraction

Biopsy nodule samples were cut with a scalpel into small pieces. Pieces were macerated with pestle and mortar, then transferred to sterile tubes with 10 ml sterile phosphate-buffered saline (PBS) to prepare tissue homogenates. Tubes were centrifuged at 1000 g, and 200 μl of supernatant was transferred to an Eppendorf tube for DNA extraction.

DNA extraction from skin samples was performed using DNeasy Blood & Tissuekit (Qiagen, Germany) according to the manufacturer’s recommendations. The DNA was eluted using 70 ul elution buffer and stored at − 20 °C until further use.

### Molecular detection 

A conventional PCR was carried out to amplify a 390 bp fragment within the P32 gene of capripoxviruses [28]. The PCR was performed using the Platinum™ Taq DNA Polymerase kit (cat# 10966–026) in a reaction volume of 25 μl containing 2.5 μl 10 X PCR buffer, 0.75 μl Magnesium chloride (50 mM), 0.5 ul dNTPs (10 mM), 0.1 μl Platinum Taq DNA polymerase, 400 nM of each primer and 1 μl template DNA. The PCR tubes containing the above mixture were transferred into a thermal cycler (T-1000, Bio-Rad, USA), and amplification was conducted with the following program: initial denaturation at 94 °C for 5 min, 38 cycles denaturation at 94 °C for 30 s, annealing at 50 °C for 30 s, extension at 72 °C for 30 s; and a final extension phase run at 72 °C for 5 min.

The PCR products were separated by gel electrophoresis on a 1.5% agarose for 60 min and visualized with a gel documentation system (UVP GelStudio PLUS Gel Documentation Imaging Systems, Analytik Jena, Germany).

A real-time PCR for the detection of capripoxvirus DNA was performed as previously described [31] with some modifications.

Briefly, the PCR mixture was set up in a reaction volume of 25 μl, containing 12.5 μl of the iQsupermix (Bio-Rad, USA), 400 nM of each primer, 250 nM of the fluorogenic probe and 5 μl of template. The reaction consisted of an initial denaturation step at 95 °C for 10 min, followed by 45 cycles at 95 °C for 15 s and 60 °C for 60 s with the fluorescence recording at the end of the combined annealing elongation step. The assaywasperformed using the CFX real-time PCR detection system (Bio-Rad).

### Amplification and sequencing of the RPO30, GPCR, and EEV glycoprotein genes

The RPO30 and the GPCR were amplified as previously described [19].

A pair of primers; EEVGly F- 5′- ATGGGAATAGTATCTGTTGTATACG-3′ and EEVGly R-5′- CGAACCCCTATTTACTTGAGAA-3′ were designed for the amplification of fragments containing the partial EEV glycoprotein (encoded by ORF LSDV126) and hypothetical protein LSDV 127 gene [18]. The PCR reaction was performed in a reaction volume of 20 μl containing 500 nM of each of the forward and reverse primers, 0.2 mM of dNTPs, 1x buffer (Qiagen), 2.5 U of Taq DNA polymerase (Qiagen), and 2 μl template DNA. The amplification consisted of an initial denaturation at 95 °C for 4 min followed by 35 cycles of 95 °C for 40 s, 55 °C for 30 s, and 72 °C for 1 min, and a final extension step at 72 °C for 7 min.

The PCR products were separated by electrophoresis on a 1.5% agarose gel at 100 V for 60 min and visualized using a Gel Documentation System (Bio-Rad, USA).

The PCR amplicons were purified using the Wizard SV Gel and PCR clean-up system kit (Promega) according to the manufacturer’s instructions. LGC Genomics (Germany) performed the sequencing of the purified PCR amplicons. Vector NTI 11.5 software (Invitrogen, USA) was used for sequencing data analysis and assembly.

### Phylogenetic analysis

Nucleotide sequences were aligned using the Muscle algorithm and the codon option implemented in MEGA7 [42]. The complete RPO30 and GPCR gene sequences of additional CaPVs (LSDVs, GTPVs, and SPPVs), retrieved from GenBank, were included for comparative analyses.

The file with aligned sequences in FASTA was converted to a Nexus format using Seaview. The Bayesian phylogenetic inference was performed with BEAST v1.8.4 [43]. First, the BEAUti module was used to generate BEAST files using the HKY substitution +G nucleotide substitution and a UPGMA starting tree option. The Markov Chain Monte Carlo method was run with BEAST, for 10,000,000 generations with a sample taken each 10,000 generations. The TRACER program was used to inspect the log files and determine the optimum number of burn-in based on the Effective Sample Sizes (ESS > 200).

TreeAnnotator was used to generate the Maximum Clade Credibility (MCC) after discarding the 3% burn-in. The tree was visualized with the associated meta-data using the ggtree package in R [44]. Additionally, for the GPCR tree, the multiple sequence alignment file of the nucleotide sequences was imported. A specific slice of the alignment, between positions 80 and 120, was visualized together with the tree [44].

## Supplementary Information

**Additional file 1.**

## Data Availability

DNA sequences generated and analyzed under the current study are available in GenBank under accession numbers MT448690 to MT448701. All the remaining datasets generated during this study are available from the corresponding author on request.

## Abbreviations

CDLI: Central Disease Investigation Laboratory
DNA: Deoxyribonucleic acid
dNTP: Deoxynucleotide triphosphate
EEV: Extracellular enveloped virus
ESS: Effective Sample Sizes
GPCR: G protein-coupled receptor
GTPV: Goatpox virus
HRM: High-resolution melting
LSD: Lumpy skin disease
LSDV: Lumpy skin disease virus
MCC: Maximum clade credibility
OIE: World Organization for Animal Health
ORF: Open reading frame
PBS: Phosphate-buffered saline
PCR: Polymerase chain reaction
RPO30: RNA polymerase 30 kDa subunit
SPPV: Sheeppox virus
