# Supplementary material for: Molecular characterization of lumpy skin disease virus (LSDV) emerged in Bangladesh reveals unique genetic features compared to contemporary field strains
Source: BMC Vet Res. 2021 Jan 29;17:61. doi: 10.1186/s12917-021-02751-x (PMC7844896; doi:10.1186/s12917-021-02751-x)
Supplement: Supplementary file 1 — Additional file 1. [file 12917_2021_2751_MOESM1_ESM.pdf]

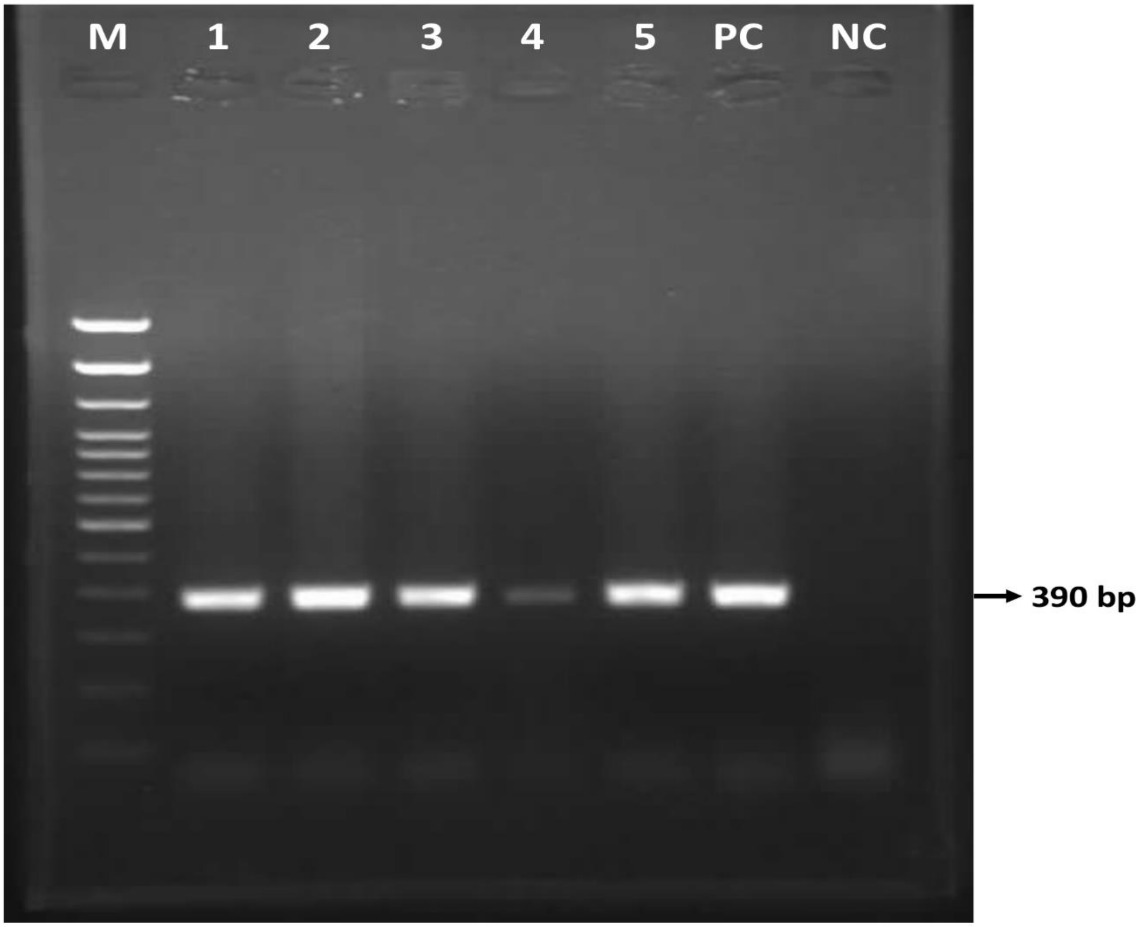

Figure 2. Agarose gel electrophoresis showing the 390 bp amplicon of P32 gene for selected samples of Bangladesh. Lane M: 100 bp DNA ladder, Lane 1-5: LSDV field samples, Lane PC: positive control, lane NC: negative control
